# The magnetosome model: insights into the mechanisms of bacterial biomineralization

**DOI:** 10.3389/fmicb.2013.00352

**Published:** 2013-11-26

**Authors:** Lilah Rahn-Lee, Arash Komeili

**Affiliations:** Plant and Microbial Biology, University of California BerkeleyBerkeley, CA, USA

**Keywords:** intracellular biomineralization, magnetotactic bacteria, bioremediation, magnetite, selenium nanospheres, cadmium sulfide, calcium carbonate, nanoparticles

## Abstract

Though the most ready example of biomineralization is the calcium phosphate of vertebrate bones and teeth, many bacteria are capable of creating biominerals inside their cells. Because of the diversity of these organisms and the minerals they produce, their study may reveal aspects of the fundamental mechanisms of biomineralization in more complex organisms. The best-studied case of intracellular biomineralization in bacteria is the magnetosome, an organelle produced by a diverse group of aquatic bacteria that contains single-domain crystals of the iron oxide magnetite (Fe_3_O_4_) or the iron sulfide greigite (Fe_3_S_4_). Here, recent advances in our understanding of the mechanisms of bacterial magnetite biomineralization are discussed and used as a framework for understanding less-well studied examples, including the bacterial intracellular biomineralization of cadmium, selenium, silver, nickel, uranium, and calcium carbonate. Understanding the molecular mechanisms underlying the biological formation of these minerals will have important implications for technologies such as the fabrication of nanomaterials and the bioremediation of toxic compounds.

## INTRODUCTION

The molecules of life, sugars, lipids, and proteins, are in large measure made of only a few of the elements abundant on earth: carbon, hydrogen, nitrogen, and oxygen. Life has developed a universal economy, including the ribosome, nucleic acid polymerases, and proteases, for using and recycling these elements into new sugars, lipids and proteins. Organisms that build materials out of the remainder of the periodic table to harness the hardness, density, and unique chemistry of these diverse elements must develop whole new mechanisms for obtaining, manipulating, and incorporating them. This is called biomineralization.

The most ready examples of biomineralization are macroscopic structures built by multicellular organisms, such as the calcium phosphate bones of vertebrates or the magnetite teeth of chitons. However, biomineralization is a widespread trait found in many single cell organisms, such as bacteria. The ease of studying bacteria, their deep branching position in the tree of life, and the variety of elements they can mineralize, including toxic pollutants, make bacterial biomineralizers an exciting area of study for those seeking to better understand mechanisms of biomineralization or looking for better chemistries to construct nanomaterials.

Unfortunately, although bacteria create a wide variety of biominerals, very little is understood mechanistically about any one case. The best studied of these by far is magnetotactic bacteria (MTB), which biomineralize crystals of the iron oxide magnetite (Fe_3_O_4_) or the iron sulfide greigite (Fe_3_S_4_) inside membrane-bound organelles called magnetosomes. These minerals have inherent magnetic properties, and it is thought that MTB exploit these magnetic properties for navigation by using the earth’s geomagnetic field to guide their search for their preferred low-oxygen environment (Frankel and Bazylinski, 2009).

**FIGURE 1:**
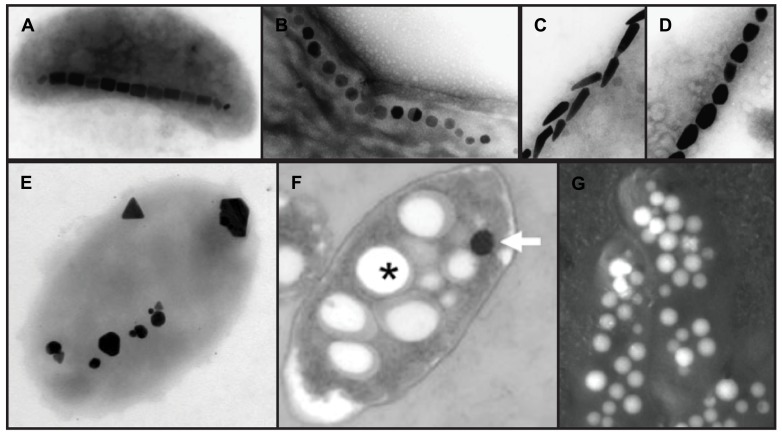
**Electron micrographs of intracellular bacterial biominerals.** **(A–D)**, magnetite crystals in different species of magnetotactic bacteria, copyright 2008 Federation of European Microbiological Societies (Schüler, 2008). **(E)**, silver minerals in *P. stutzeri*, copyright 1999, National Academy of Sciences, USA (Klaus et al., 1999). **(F)**, *T. selenatis*, copyright 2011 the authors (Debieux et al., 2011). Asterisk, Poly(3-hydroxybutyrate) granule; arrow, selenium nanosphere. **(G)**, carbonate deposits in a cyanobacterium, copyright 2012 American Association for the Advancement of Science (Couradeau et al., 2012). In this backscatter scanning electron micrograph, electron dense carbonate deposits appear white. Images are not to scale.

There is huge diversity in the size and shape of magnetite or greigite nanocrystals produced by different species of MTB (Lins et al., 2000; Faivre and Schüler, 2008; Schüler, 2008, **Figure 1**). Due to their genetic tractability and ease of growth in the laboratory, two closely related alpha-proteobacteria, *Magnetospirillum magneticum* AMB-1 (AMB-1) and *M. gryphiswaldense* MSR-1 (MSR-1), have been the model systems for the mechanistic understanding of biomineralization. These organisms both produce cubo-octohedral magnetite crystals. The current picture of the mechanisms employed by these bacteria to biomineralize magnetite are covered in these reviews (Jogler and Schüler, 2009; Komeili, 2012) and summarized in **Figure 3**.

Bacteria undergo intracellular biomineralization under a variety of conditions and for different purposes. In some cases, organisms are fed metals to see if they can mineralize them. For example, *Escherichia coli* and *Rhodopseudomonas palustris* have both been shown to biomineralize cadmium when it is provided to them under experimental conditions (Sweeney et al., 2004; Bai et al., 2009), and AMB-1 can mineralize tellurium nanorods separately from its magnetite crystals, creating a biomagnetic method of recovering this rare element from the environment (Tanaka et al., 2010). Other organisms are able to use biomineralization to detoxify pollutants such as nickel, uranium, or silver encountered in the environment by transforming them into less bio-accessible states (Klaus et al., 1999; Zhan et al., 2012; Sousa et al., 2013). Still others manage their own waste products with biomineralization, including photosynthesizing cyanobacteria (Couradeau et al., 2012), and selenite-respiring bacteria (Debieux et al., 2011). Finally, MTB specifically import iron from the environment in order to build an organelle that is useful to the cell.

Although they have exciting implications for nanotechnology, bioremediation, and bacterial cell biology, little is understood about the biomineralization processes described above. MTB biomineralization can serve as a model for these exotic and less well-studied cases. Here, we discuss recent advances in the study of MTB magnetite formation, and speculate about their implications for understanding the diverse array of cases of bacterial intracellular biomineralization.

## SOURCE OF THE MINERAL

Before biomineralization can take place, the raw materials must be obtained. For many bacteria, biomineralization is a way to cope with an environmental toxin or a waste product, so the main elements that form the final mineral do not need to be sourced. For example, *Pseudomonas alcaliphila* has been shown to mineralize toxic Ni(II) to Ni(0) (Zhan et al., 2012), and *Thauera selenatis* manages selenite, a waste product of its respiration on selenate, by mineralizing Se(0) nanospheres (Debieux et al., 2011). In both of these cases, the resultant mineral is elemental nickel or selenium, so no further material is needed.

Like *T. selenatis*, cyanobacteria handle carbonate, the waste product of photosynthesis, by precipitating it, in this case with cations such as calcium. Recently, a cyanobacterium from the Gloeobacterales order has been shown to perform this precipitation intracellularly, using calcium as well as the cations magnesium, strontium, and barium (Couradeau et al., 2012). Remarkably, these other cations were enriched relative to calcium in the mineral, in the case of barium over one thousand fold (Couradeau et al., 2012), suggesting an unknown mechanism for recruiting and incorporating barium into the mineral.

In some cases there are good guesses, if no hard evidence, of the source of co-precipitating elements. For example, *P. stutzeri* survives in toxic concentrations of silver by producing large, periplasmic silver crystals, some in the form of the silver sulfide acanthite (Klaus et al., 1999). The authors of this study hypothesize that the sulfur may be coming from hydrogen sulfide gas, which *P. stutzeri* is known to produce (Slawson et al., 1994; Klaus et al., 1999). A sulfur source is similarly needed for *E. coli* and *R. palustris* to produce cadmium sulfide (CdS) when grown at high cadmium concentrations. Cysteine has been shown to act as a sulfur source during extracellular CdS precipitation (Wang et al., 2000), and indeed the activity of cysteine desulfhydrases, enzymes that remove sulfide from the amino acid cysteine, were found enriched in the cellular fraction of *R. palustris* where biomineralization occurs, and the levels of these enzymes were found to rise later in the cell cycle, during maximum CdS production (Bai et al., 2009).

MTB are different from other biomineralizers in that they need to import iron to make magnetite. However, an understanding of MTB iron transport could elucidate the import strategies of co-precipitating elements for other bacteria. Early evidence suggested that MTB could produce siderophores, raising the possibility that they could import insoluble ferric iron (Paoletti and Blakemore, 1986). Partial support for this model comes from the examination of a non-magnetic AMB-1 mutant that appears to have defects in siderophore-mediated iron uptake under simulated iron starvation conditions (Calugay et al., 2004). However, careful analysis of siderophore production by AMB-1 has found that these molecules are made as a result of iron depletion following magnetite production, making it unlikely that they are a central element of biomineralization in MTB (Calugay et al., 2003).

Recent studies in MSR-1 suggest that at least some of the iron transport for magnetite synthesis occurs through two copies of the ferrous iron transporter FeoB. The *feo* iron transport genes are common to most bacteria, including all MTB investigated so far, many of which contain additional, magnetosome-specific copies of *feoB* (Lefèvre et al., 2013). Deletions of *feoB1* (Rong et al., 2008) and *feoB2* (Rong et al., 2012) reduce the magnetite content of MSR-1, as does a deletion of *fur*, which encodes an iron response regulator that effects the transcription of both *feoB* genes (Uebe et al., 2010; Qi et al., 2012).

Early transposon mutagenesis of AMB-1 (Matsunaga et al., 1992) yielded a disruption in a gene named *magA* that appeared to be transcriptionally regulated by iron and caused the accumulation of iron when heterologously expressed in *E. coli*-derived membrane vesicles (Nakamura et al., 1995). Indeed, when expressed in mammalian cell culture, *magA* appears to increase cellular iron content (Goldhawk et al., 2009) and cause the formation of small iron deposits (Zurkiya et al., 2008). Recently, however, in-frame deletions have been made of *magA* in both AMB-1 and MSR-1, and no biomineralization phenotype was observed (Uebe et al., 2012), leaving the FeoB proteins as the only factors that have been clearly demonstrated to be involved in iron uptake for magnetite formation.

Much is still unknown about MTB iron transport. A double *feoB* deletion is still able to biomineralize some magnetite (Rong et al., 2012), suggesting that other transport systems exist. The magnetosome proteins MamM and MamB, which are members of the cation diffusion facilitator family of metal transporters (Murat et al., 2010; Uebe et al., 2011), and MamZ and MamH, which are members of the major facilitator superfamily of transporters (Raschdorf et al., 2013), have been proposed as additional iron transporters for magnetite biomineralization. Although loss of these genes results in a defect in magnetic particle formation, it has not yet been shown whether or not they are transporting iron for biomineralization.

Magnetite crystals are built inside a membrane-bound compartment, and it remains to be elucidated if iron is transported through the cytoplasm, or directly from the periplasm to the compartment, as suggested by Faivre et al. (2007). Transport systems including FeoB could act at any of these steps (**Figure 3**). Whatever the mechanism of iron import, MTB must also source the oxygen for magnetite. O_2_ from air is a tempting guess, and early speculation focused on whether there was a competition for oxygen between biomineralization and respiration (Blakemore et al., 1985). However, isotope analysis demonstrates that the oxygen in MTB-biomineralized magnetite comes from water (Mandernack et al., 1999). This is a good reminder that the obvious source of the raw materials for biomineralization may not be the source that the bacteria actually use.

## CHEMISTRY

Once soluble minerals are obtained, they must be manipulated chemically to become insoluble precipitates or crystal deposits. This can happen simply by rearranging chemical bonds, for example soluble uranium VI is precipitated as the insoluble U(VI)-containing uranium phosphate meta-autunite during bioremediation in *Rhodanobacter* A2-61 (Sousa et al., 2013). More often, however, the redox state of the mineral is altered. In many of the cases discussed here, glutathione and other thiols, central players in cellular redox homeostasis, are thought to play key roles in the mineralization process (Sweeney et al., 2004; Debieux et al., 2011).

One of the major redox pathways in microaerophilic or anaerobic bacteria such as MTB is the denitrification pathway, which reduces nitrate to nitrogen gas for respiration. Recently, MSR-1 has been shown to possess a complete denitrifictaion pathway and the first step, the *nap* genes whose products catalyze the reduction of nitrate (NO_3_) to nitrite (NO_2_), was shown to be essential for growth without oxygen (Li et al., 2012). Intriguingly, deletions of both *nap* genes and *nir* genes, whose products catalyze the reduction of nitrite to nitric oxide (NO), have biomineralization defects (Li et al., 2012, 2013). Deletions of these genes also disrupt respiration and growth, presumably with pleotropic effects, making interpretation difficult. However, it is tempting to imagine that they are regulating the redox state of the cell, or perhaps even oxidizing ferrous iron directly in order to allow for magnetite biomineralization (Li et al., 2012, 2013). Indeed, a nitrite reductase from *M. magnetotacticum* MS-1 has been shown to oxidize Fe(II) *in vitro* (Yamazaki et al., 1995). Since denitrification genes are common among bacteria, they could also play a role in other biomineralization mechanisms.

MTB also contain a dedicated set of redox active proteins. Bioinformatic analysis identified the genes *mamE*, *mamP*, and *mamT* as common to MTB, with their products sharing a unique configuration of two closely spaced CXXCH heme-binding motifs, termed the magnetochrome domain (Siponen et al., 2012). Purified MamP and MamE have spectral and redox characteristics consistent with c-type cytochromes (Siponen et al., 2012). Deletions of these genes (Murat et al., 2010; Quinlan et al., 2011) or of *mamX*, another magnetochrome gene (Raschdorf et al., 2013; Yang et al., 2013), result in biominerlization defects, as do point mutations in the putative heme-binding CXXCH motif (Quinlan et al., 2011; Raschdorf et al., 2013). Since some of these genes also contain protein-interaction PDZ motifs, it has been suggested that they form a protein complex that serves as an electron transport chain to regulate electron flow for biomineralization (Siponen et al., 2012).

**FIGURE 2:**
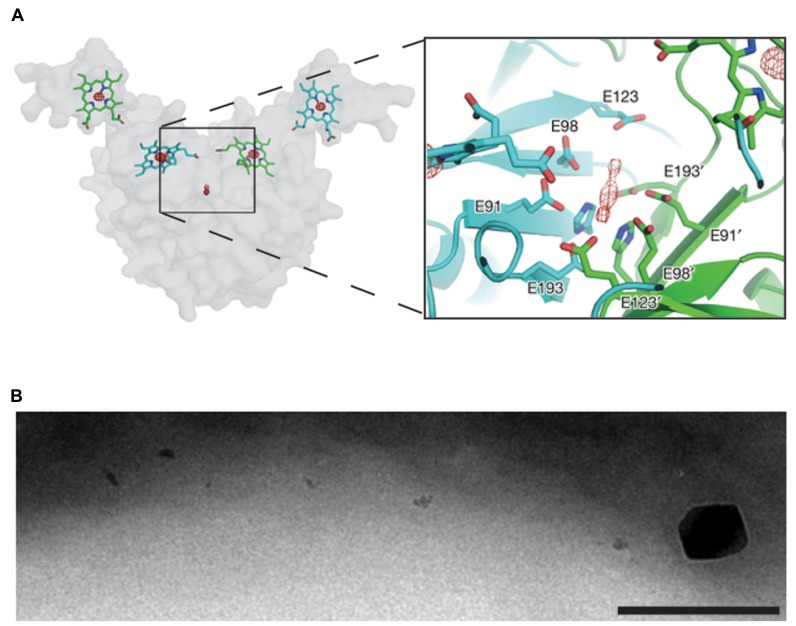
**MamP oxidizes iron and regulates magnetite crystal size.** **(A)**, the crystal structure of MO-1 MamP with two atoms of iron bound in the acidic pocket, adapted by permission from Macmillan Publishers Ltd: Nature Siponen et al., 2013, copyright 2013. **(B)**, a *mamP* mutant in AMB-1 with many small magnetite crystals and rare large ones. Scale bar 200 nm.

The recent crystal structure of the magnetochrome protein MamP from strain MO-1 provides our first mechanistic insight into this new class of cytochromes (Siponen et al., 2013). This structure suggests that the functional unit of MamP is a dimer, with four heme groups surrounding a central acidic pocket. Incubating the MamP crystal with Fe(II) before imaging resulted in a new density in the acidic pocket, potentially two atoms of iron (**Figure 2**). To demonstrate that the acidic pocket is important for function, a *mamP* deletion strain of AMB-1 was complemented either with wild type *mamP* or a mutant gene with the acidic residues changed to alanine. The allele lacking the acidic residues failed to complement even though it was expressed, suggesting the chemical nature of this pocket is important for MamP function. When MO-1 MamP was incubated with Fe(II) *in vitro* first ferrihydrite, which contains Fe(III), and then magnetite, which contains both Fe(II) and Fe(III), were formed, suggesting that MamP bound and then oxidized the iron. As shown in **Figure 2**, in AMB-1 a *mamP* mutant has many small crystals with rare crystals that are larger than normal (Murat et al., 2010). Perhaps inability to regulate the redox state of iron can cause unchecked mineral growth.

Many bacterial biomineralizers need to control the redox state of their minerals. It will be interesting to see if the others, like MTB, have dedicated electron transport chains or redox-active proteins for this purpose. *T. selenatis*, which respires selenate and biomineralizes selenium, uses its selenate reductases in this process (Debieux et al., 2011), but as its product of respiration is incorporated into the biomineral itself, it is a unique case. MTB also employ general respiration reductases for magentosome formation. Perhaps this is a common feature of bacterial biomineralization.

## REGULATION OF MINERAL STATE

In some cases of intracellular bacterial biomineralization the type of mineral or crystal phase produced is not strictly controlled. For example, the cyanobacterial carbonate deposits, while similar to the mineral benstonite, have an unusual stoichiometry and are amorphous (Couradeau et al., 2012). *P. stutzeri* and *Rhodanobacter* A2-61 each build three different kinds of crystalline silver or uranium phosphate minerals, respectively (Klaus et al., 1999; Sousa et al., 2013). Other bacteria, however, produce uniform crystalline minerals, suggesting tight regulation over the mineralization process. The CdS crystals produced by *R. palustris* or *E. coli*, for example, are uniform cubic or hexagonal CdS, respectively (Sweeney et al., 2004; Bai et al., 2009). The elemental selenium deposits produced by *T. selenatis* are also uniform (Debieux et al., 2011). No mechanisms are known that regulate the mineral forms in these cases.

For guidance, we can turn to MTB. Some MTB crystalize magnetite, and others greigite. Which mineral is produced is species-specific, suggesting there is genetic control of mineral state. Indeed, a bacterium, *Candidatus* Desulfamplus magnetomortis BW-1, was recently isolated that can mineralize both. Genomic analysis suggests that this strain possesses two sets of genes for biomineralization, one that is more closely related to those of magnetite-producers, and the other to greigite-producers (Lefèvre et al., 2011). BW-1 produces either magnetite or greigite crystals depending on the chemical environment (Lefèvre et al., 2011), though whether this phenomenon is chemical in nature or whether the cell is responding to the environment either genetically or biochemically remains unknown.

Some MTB genes have been shown to affect the mineral state. Although magnetite or greigite are invariably produced in wild type MTB, recent studies in MSR-1 have uncovered mutant backgrounds in which some of the mineral produced is hematite, an Fe(III)-containing iron oxide (Fe_2_O_3_). These include the deletion of *mamX*, the PDZ-containing magnetochrome gene described above, or of *mamZ*, a gene with homology to iron reductases and transporters (Raschdorf et al., 2013). In addition, a point mutation in the metal-binding site of MamM, a putative iron transporter, resulted in hematite crystals in MSR-1 (Uebe et al., 2011). Taken together, these results suggest that the abilities of cells to correctly regulate the flow of iron and its redox state are crucial to their ability to direct the biomineralization of iron toward magnetite.

These results are consistent with recent studies of the early stages of magnetite formation in AMB-1 (Baumgartner et al., 2013) and MSR-1 (Fdez-Gubieda et al., 2013), which show that iron transforms from a phosphate-rich ferric hydroxide, potentially the previously observed ferritin (Faivre et al., 2007), through a ferrihydrite intermediate, into magnetite (Baumgartner et al., 2013; Fdez-Gubieda et al., 2013). Rarely, small hematite crystals were observed, consistent with previous findings that young magnetite crystals are surrounded by a layer of hematite (Staniland et al., 2007). Ferrihydrite is an iron oxide (Fe_2_O_3_) coordinated with water. Ferrihydrite and small crystals of hematite both contain Fe(III) and are thought to be unstable enough to transform into the mixed-valence magnetite (Baumgartner et al., 2013), making them attractive intermediates in this process. Perhaps, in mutants where iron redox metabolism is disturbed, some of the transitional hematite cannot be transformed into magnetite, and is able to grow to a stable size. These studies highlight the importance of redox control to the ability of MTB and other bacteria to regulate the type of minerals they produce.

## REGULATION OF MINERAL SIZE AND SHAPE

Some cases of bacterial biomineralization produce crystals of relatively uniform size and shape, while others are not so well controlled. For example, the CdS nanoparticles produced by *R. palustris* are spherical and have an average diameter of 8 nm, with very few crystals outside the 6–9 nm rage (Bai et al., 2009). It is unclear if there is any active mechanism to regulate their size. However, similar crystals produced in the genetically distinct *E. coli* are also small and uniform in size, from 2 to 5 nm (Sweeney et al., 2004), suggesting that under the conditions present inside these bacterial cells CdS crystals cannot get very large. In contrast, the silver crystals produced by *P. stutzeri* vary remarkably in size and shape (Klaus et al., 1999). Some of these crystals are round or small, while others are polygonal or triangular, and some take up a large portion of the cellular space (**Figure 1**). Clearly the conditions keeping CdS crystals small and uniform in *R. palustris* and *E. coli* are not acting on the silver crystals in *P. stutzeri*.

**FIGURE 3:**
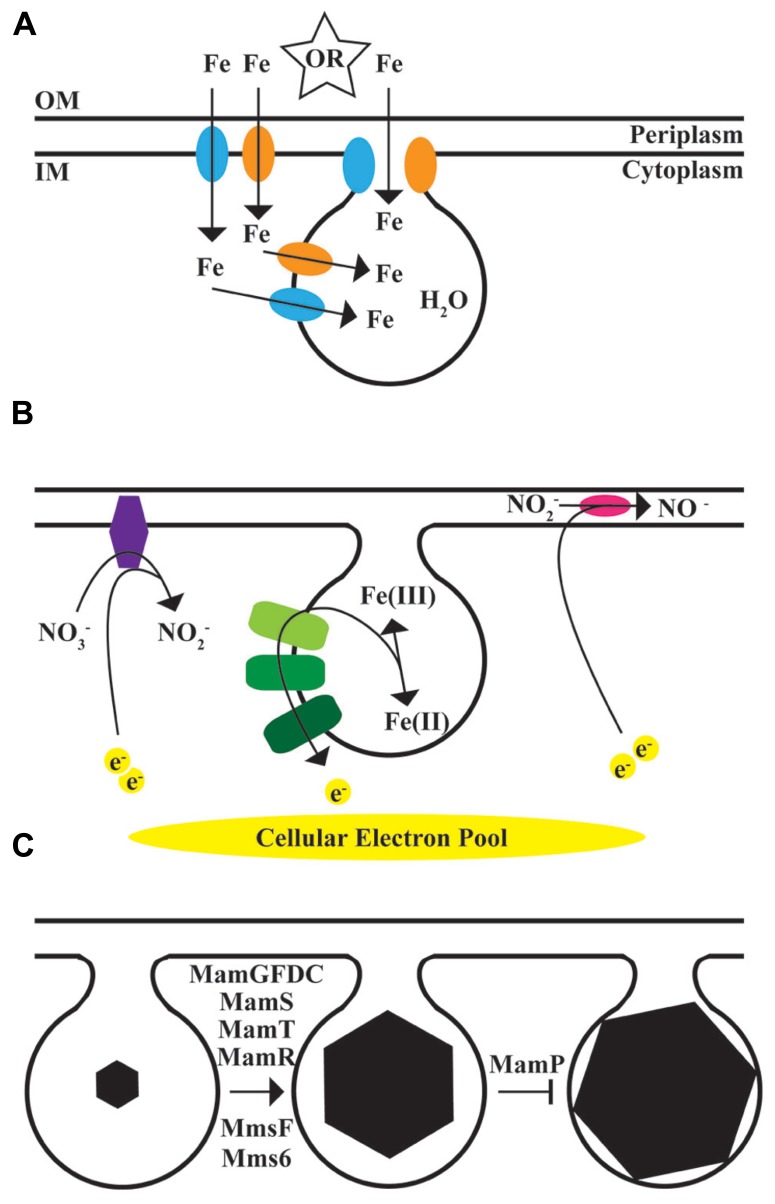
**The magnetosome model.** Biomineralization in MTB occurs within a membrane compartment derived from the inner membrane. **(A)**, Source of the mineral. Iron transporters, including FeoB1, blue, and FeoB2, orange, transport iron for magnetite synthesis. Iron either comes through the inner membrane into the cytoplasm and then through the magnetosome membrane into the compartment, or is transported directly from the periplasm to the magnetosome. Other unidentified transport systems are expected to be involved. Water is the source for the oxygen. OM, outer membrane; IM, inner membrane. **(B)**, Chemistry and Regulation of mineral state. Members of the magnetochrome transport chain, in green, transport electrons to or from iron. Nap, purple, and Nir, magenta, use electrons to reduce nitrate or nitrite. **(C)**, Regulation of mineral size and shape. In addition to mms6, described here, mamGFDC (Scheffel et al., 2008), mamS, mamT, and mamR (Murat et al., 2010) as well as mmsF (Murat et al., 2012) have all been shown to promote the growth of magnetite crystals to the correct shape and size. MamP appears to limit crystals from growing too large (Murat et al., 2010).

MTB build magnetite crystals to sense and respond to magnetic fields (Frankel and Bazylinski, 2009), and crystal size and shape can greatly affect their ability to perform this task. Diverse MTB produce magnetite crystals of different sizes and shapes (**Figure 1**), presumably fine-tuned for the performance needs of each individual organism. We have some clues as to the genetic factors that control the size and shape of AMB-1 and MSR-1 magnetite crystals (Scheffel et al., 2008; Ding et al., 2010; Murat et al., 2010; Lohsse et al., 2011; Quinlan et al., 2011; Uebe et al., 2011; Murat et al., 2012), which are reviewed in **Figure 3**.

One of these factors that specifically regulate the mineralization of iron is Mms6, a small acidic protein that was identified because it is closely associated with magnetite crystals isolated from AMB-1 (Arakaki et al., 2003). *In vitro* magnetite synthesis reactions produce a wide range of crystal sizes and shapes, but the addition of Mms6 results in a uniform range of crystal sizes and shapes that look similar to crystals made by bacteria *in vivo* (Arakaki et al., 2003, 2010; Amemiya et al., 2007; Prozorov et al., 2007; Galloway et al., 2011). Though short, the Mms6 sequence has a number of interesting features, including an amphipathic character, with a hydrophobic N-terminal domain and hydrophilic C-terminal domain. Recent work suggests that these features allow Mms6 monomers to assemble into micelles with the iron-binding C-termini of the monomers facing outward (Wang et al., 2012; Feng et al., 2013). Upon binding of iron, the Mms6 monomers in the micelle undergo a structural change, which may be important to their function in regulating the mineralization of magnetite (Feng et al., 2013).

Similar conceptually, though not biochemically, to Mms6, a protein named SefA has been isolated from *T. selenatis* that can regulate the precipitation of selenium *in vitro* (Debieux et al., 2011). SefA, which is conserved among a few bacteria known to respire selenate, was identified via its association with the selenium nanospheres that are secreted from *T. selenatis* during respiration on selenite. The strongest evidence that SefA may fulfill a role similar to Mms6 is data from *ex vivo* and *in vitro* experiments. *E. coli* that are grown in the presence of selenite are able to produce small 50 nm selenium deposits inside their cells. However, when SefA is expressed, 300 nm selenium deposits are produced, and the *E. coli* cells are tolerant to growth in higher selenite concentrations (Debieux et al., 2011). Selenite can be reduced *in vitro* to selenium. In the absence of SefA, an amorphous precipitate is created, but if purified SefA protein is included in the reaction, 300 nm selenium nanospheres are produced (Debieux et al., 2011).

Little is known about how the shape or size of biominerals are regulated in other bacterial systems, even for MTB outside of the AMB-1/MSR-1 group of alpha proteobacteria. Potential clues may come from recent genomic work that has identified a group of 29 genes, termed *mad* genes, for magnetosome associated delta proteobacteria, that are conserved among the delta proteobacterial MTB (Lefèvre et al., 2013). Unlike the alpha proteobacteria, these MTBs build elongated, bullet-shaped crystals and do not have the shape-determining factor Mms6. For one of these organisms, *Desulfovibrio magneticus* RS-1, proteomics work has identified crystal-associated proteins (Matsunaga et al., 2009). Some of these are coded for by the recently identified *mad* genes *mad25* (*DMR_40870*), *mad23* (*DMR_40890*), *mad10* (*DMR_40950*), *mad11* (*DMR_40960*), and *DMR_41300*, which has homology to *mad12*, although it was not identified by Lefèvre et al. (2013). It will be exciting to see if any of these candidates prove to be a shape-determining factor for bullet shaped crystals.

The examples of Mms6 and SefA suggest that at least in some cases bacteria that have evolved biomineralization systems can regulate the size and shape of their minerals with proteins that have intrinsic properties to bind the mineral and are able to perform at least some of their regulatory functions *in vitro*, away from the rest of the biomineralization machinery. Those seeking the regulators of mineral size and shape in other bacterial systems might consider looking for proteins that remain closely associated with the mineral after isolation from the bacteria and testing those proteins for *in vitro* function.

## CONCLUSION

The reasons for and conditions under which bacteria produce intracellular biominerals are broad and varied. Intracellular, as opposed to extracellular, biomineralization has diverse effects on the cell, including changing cellular pH (Couradeau et al., 2012), buoyancy (Bai et al., 2009; Couradeau et al., 2012), and susceptibility to reactive oxygen species (Guo et al., 2012). Nanomaterials produced in this way have the advantage that their make-up and shape are controlled genetically and they are often surrounded by an organic layer that can aid in dispersal or be modified to carry payloads such as antibodies (Bai et al., 2009; Pollithy et al., 2011). As we discover more of these cases, the magnetosome model can serve as our guide to understanding mechanisms behind the formation of bacterial intracellular biominerals.

## Conflict of Interest Statement

The authors declare that the research was conducted in the absence of any commercial or financial relationships that could be construed as a potential conflict of interest.
